# Usutu Virus in Migratory Song Thrushes, Spain

**DOI:** 10.3201/eid1907.130199

**Published:** 2013-07

**Authors:** Ursula Höfle, Virginia Gamino, Isabel G. Fernández de Mera, Atilio J. Mangold, José-Antonio Ortíz, José de la Fuente

**Affiliations:** Instituto de Investigación en Recursos Cinegéticos (IREC)-CSIC-UCLM-JCCM, Ciudad Real, Spain (U. Höfle, V. Gamino, I.G. Fernández de Mera, J. de la Fuente);; Estación Experimental Agropecuaria Rafaela, Santa Fe, Argentina (A.J. Mangold);; Universidad Complutense de Madrid, Madrid, Spain (I.G. Fernández de Mera);; Medianilla S.l. Benalup, Cádiz, Spain (J.A. Ortiz);; Oklahoma State University, Stillwater, Oklahoma, USA (J. de la Fuente)

**Keywords:** Usutu virus, song thrush, migratory bird, encephalitis, viruses

**To the Editor:** Usutu virus (USUV), a member of the Japanese encephalitis virus antigenic group, was first detected in 1959 in mosquitoes in South Africa (*1*), and it emerged in 1996 in blackbirds (*Turdus merula*) in Italy (*2*). Recent cases of USUV infection in asymptomatic blood donors (*3*) and severe disease in immunocompromised persons (*4*) have shown its zoonotic potential.

Epidemiology and molecular phylogeny of USUV isolated in Italy, Austria, Hungary, Switzerland, and Germany suggest that stable endemic mosquito–bird cycles have been established in Europe (*5*,*6*). Where active vector surveillance programs exist, USUV is detected in mosquitoes before bird deaths and human infections. USUV strains similar to African strains were detected in mosquitoes in Spain in 2006 and 2009 (*7*,*8*).

In November 2012, two live song thrushes (*Turdus philomelos*) with central nervous system signs were recovered from a die-off of ≈10 birds at a hunting estate in southern Spain. A full necropsy was conducted on the 2 thrushes (which died shortly after capture), and samples were collected for virus detection and histopathologic examination. Total RNA was extracted from oral and cloacal swab specimens, from serum from a cardiac blood clot, and from heart, kidney, spleen, and brain tissues by using High Pure RNA Tissue Kit (Roche Diagnostics, Barcelona, Spain) and analyzed by generic flavivirus SYBR Green (QIAGEN, Madrid, Spain) real-time reverse transcription PCR (RT-PCR) and by a generic conventional nested flavivirus RT-PCR (*9*). The product of the first PCR (1,048 bp) was resin purified, cloned into pGEM-T (Promega, WI, USA), and sequenced. This sequence was compared with sequences of European and African USUV strains that were available in GenBank. In addition to the histopathologic examination, we used a polyclonal primary rabbit antibody directed against West Nile virus with proven cross-reactivity to other flaviviruses for viral antigen detection by immunohistochemical testing (*9*).

The thrushes were an adult male and female in poor body condition; they had greenish urate-soiled feathers around the cloaca. Subcutaneous or visceral fat deposits were absent, and the pectoral muscle was partially atrophied, more severely in the male. Both birds showed severe generalized congestion.

The serum, brain, and pool of cloacal and oropharyngeal swab specimens of both birds yielded a strongly positive signal in the generic flavivirus real time RT-PCR. Sequencing of the PCR product obtained in the generic flavivirus RT-PCR (GenBank accession no. KC437386) showed a 96%–97% homology to published USUV sequences. Nucleotide sequence analysis revealed a higher homology to Northern European strains (97% to BH65/11-02-03 [HE599647] and Meise H, Germany [JQ219843]; Budapest, Hungary [EF206350]; Italy 2009 [JF266698]; and Vienna 2001, Austria [AY453411]) than to a USUV strain isolated in South Africa (96% to SAAR-1776 [AY453412]). This finding was also supported by phylogenetic analysis of USUV strains (Figure, panel A), with similar results for maximum likelihood and neighbor-joining methods (data not shown).

**Figure:**
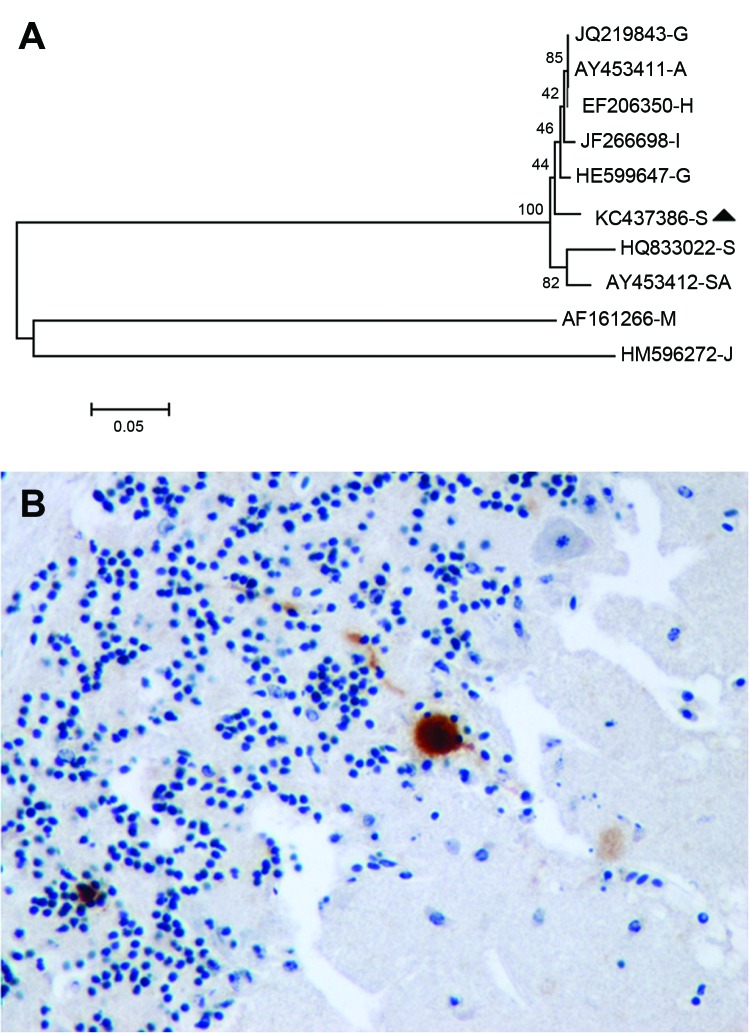
Usutu virus (USUV) in migratory song thrushes in Spain. A) Phylogenetic analysis of European and African USUV strains. The evolutionary history was inferred using the neighbor-Joining method. The optimal tree with the sum of branch length = 1.18014408 is shown. The percentages of replicate trees in which the associated taxa clustered together in the bootstrap test (1,000 replicates) are shown next to the branches. The tree is drawn to scale, with branch lengths in the same units as those of the evolutionary distances used to infer the phylogenetic tree. The evolutionary distances were computed by using the maximum composite likelihood method and are in the units of the number of base substitutions per site. The analysis involved 10-nt sequences. All ambiguous positions were removed for each sequence pair. There were a total of 929 positions in the final dataset. Evolutionary analyses were conducted in MEGA5 (www.megasoftware.net). USUV strains are identified by their GenBank accession numbers and a capital letter indicating the country of origin (A, Austria; G, Germany; I, Italy; H, Hungary; S, Spain; SA, South Africa); the sequences of the last 2 branches correspond to outgroup viruses used to root the tree (M, Murray Valley encephalitis virus [GenBank AF161266]; J, Japanese encephalitis virus [GenBank HM596272]). The song thrush strain from Spain 2012 is highlighted with a black triangle. Scale bar indicates nucleotide changes per site. B) Immunohistochemical staining with cross-reacting antibody showing USUV antigen labeling in a Purkinje cell of the cerebellum of a song thrush that died from encephalitis. Original magnification x400.

Histologically, both birds had severe encephalitis characterized by congestion, neuronal and Purkinje cell necrosis, gliosis, satellitosis, neuronophagia, and endothelial cell swelling and vasculitis. Other lesions included multiorgan congestion, necrosis of renal tubular epithelium, and moderate hemosiderosis in the liver and spleen. Intravascular cross-sections of filarial parasites were detected in pulmonary capillaries. Antigen labeling was detected in neurons in the cerebral hemispheres and brain stem and in glia cells throughout the brain (Figure, panel B). Rare Purkinje cells and neurons in peripheral ganglia (e.g., of the gizzard) as well as cardiac myofibers and renal tubular epithelial cells were positive for viral antigen.

The molecular genetic analysis, histopathologic examination, and immunohistochemical testing confirmed encephalitis caused by USUV in 2 migratory wintering song thrushes who died during a small mortality event in southern Spain. Phylogenetic analysis showed that the USUV strain infecting the diseased birds was more similar to USUV strains from Austria, Hungary, and Germany than to USUV strains isolated from mosquitoes in Spain (*7*,*8*) or on the African continent. This result, together with the fact that song thrushes are a nonresident, migratory, wintering bird species in southern Spain (*10*), provides circumstantial evidence of USUV introduction into Spain during bird migration from northern Europe. Persistence of USUV in song thrushes and recrudescence of the infection during southward migration with concomitant filarial infection, together with the presence of USUV in a local endemic cycle from previous introductions, high vector abundance, and high viral loads in infectious mosquitoes, are possible scenarios that caused this outbreak.

Our data imply that introduction of USUV (and potentially other flaviviruses such as West Nile virus lineage 2, which has not yet been detected in Spain) from Northern Europe, in addition to local endemicity and introduction from Africa, occurs, and that the zoonotic European USUV strain may be co-circulating with strains of African origin. At this time, it is not clear whether USUV strains of Spanish/African lineage differ in virulence for humans from strains from the European/African lineage. However, virus introduction by northern migrants, in combination with locally favorable conditions for vector populations, implies a risk for virus amplification and transmission and disease outbreaks in humans and horses outside the currently established mosquito-trapping period (May–October) of targeted flavivirus surveillance programs.
